# Modeling of the Response of Hydrogen Bond Properties on an External Electric Field: Geometry, NMR Chemical Shift, Spin-Spin Scalar Coupling

**DOI:** 10.3390/molecules26164967

**Published:** 2021-08-18

**Authors:** Ilya G. Shenderovich, Gleb S. Denisov

**Affiliations:** 1 Institute of Organic Chemistry, University of Regensburg, Universitaetstrasse 31, 93053 Regensburg, Germany; 2 Department of Physics, St. Petersburg State University, 198504 St. Petersburg, Russia; gldenisov@yandex.ru

**Keywords:** cyanide, hydrogen bonding, non-covalent interactions, NMR, dissociation, scalar coupling, DFT, GIAO

## Abstract

The response of the geometric and NMR properties of molecular systems to an external electric field has been studied theoretically in a wide field range. It has been shown that this adduct under field approach can be used to model the geometric and spectral changes experienced by molecular systems in polar media if the system in question has one and only one bond, the polarizability of which significantly exceeds the polarizability of other bonds. If this requirement is met, then it becomes possible to model even extreme cases, for example, proton dissociation in hydrogen halides. This requirement is fulfilled for many complexes with one hydrogen bond. For such complexes, this approach can be used to facilitate a detailed analysis of spectral changes associated with geometric changes in the hydrogen bond. For example, in hydrogen-bonded complexes of isocyanide C≡^15^N-^1^H⋯X, ^1^J(^15^N^1^H) depends exclusively on the N-H distance, while δ(^15^N) is also slightly influenced by the nature of X.

## 1. Introduction

Correlations between spectroscopic parameters and geometric and energy properties of non-covalent interactions are the main tool for the experimental study of these interactions. The variety of such correlations is great. Some of them are general, while most are only applicable to a limited type of molecular systems.

OH, and NH vibrations can be used to characterize inter- and intramolecular hydrogen bonds [1,2,3]. The intensity of these stretching vibrations correlates strongly to the energy of the corresponding hydrogen bonds [4,5]. This correlation can be proved using the energy of an intermolecular hydrogen bond measured experimentally as the enthalpy of formation [6,7,8]. The location of hydrogen atoms in X-ray data can be evaluated using Hirshfeld atom refinement [9,10,11]. For some systems with hydrogen bonds A-H…B, accurate correlations are available between the distances A-H and H…B [12,13]. Therefore, if one of these distances is known, the other can be estimated. The N…H distance in hydrogen-bonded complexes of pyridine derivatives can be obtained from the ^15^N nuclear magnetic resonance (NMR) chemical shift of these pyridines [14,15]. Then this correlation was used to establish a correlation between the N…H distance and the ^1^H NMR chemical shift of the binding proton for such complexes [16,17]. The dependences of ^1^H and ^13^C chemical shifts on hydrogen bonding have been extensively studied. They agree with dependences obtained for other experimental and empirical parameters [18,19,20,21,22]. More about the application of these and other correlations can be found elsewhere [23,24,25,26]. 

Appropriate theoretical calculations make it possible to fully understand the relationship between different parameters of the system under study and to facilitate the search for optimal correlations [27,28,29,30,31]. Such calculations are often the only method for studying weak non-covalent interactions [32,33,34,35,36] or molecular systems with competing interactions [37,38,39,40,41,42]. However, often these calculations require a very careful selection of the systems to be examined. Accurate calculations of spectral quantities generally require large basis sets [43]. This requirement can easily conflict with the size of the systems examined [44]. A correlation requires a large set of model systems in which the studied parameter varies over a wide range. It can be very difficult to find a sufficient number of molecular systems that have the appropriate size, required properties and do not introduce other competing interactions into the system. The latter requirement is important. Only in very special cases, the considered spectral parameter depends exclusively on a certain type of interaction and the observed spectral changes are associated only with changes in one of the parameters of this interaction [45,46,47]. More often, the influence of different interactions on spectral changes can be distinguished only in the course of special studies [48,49].

In some cases, these problems can be solved using the adduct under field approach [50]. The properties of a molecular system can be gradually changed by applying an external electric field [51,52,53,54,55]. Electric fields are present in molecular systems [56,57]. These fields can cause measurable spectral changes [58,59,60,61,62] and even specific chemical reactions [63,64,65]. However, a small external electric field can be also used as a tool to exert pressure on the electron density of a non-covalent bound molecular system. In this approach, the field simulates the influence of other unknown factors causing changes in the geometry of the non-covalent interaction in question. The ability of the adduct under field approach to reproduce the experimental geometry of such molecular systems in solutions has been demonstrated for various complexes [50,66,67,68]. Nevertheless, the question remains about the direct influence of the field on spectral parameters and the geometry of covalent bonds. This issue will be analyzed here.

This work discusses three aspects of the direct effect of external electric fields on molecular systems. First, we will consider the isotropic ^15^N chemical shift, δ_iso_(^15^N), and the scalar spin-spin coupling, ^1^J(^15^N^1^H), in C≡^15^N-H⋯F^−^ and C≡^15^N-H⋯FLi hydrogen-bonded complexes as functions of an external electric field directed along the axis of molecular symmetry. If the functional dependence is the same for both complexes, then the corresponding spectral parameter depends exclusively on the N-H distance and can be used to measure this distance in any C≡^15^N-H⋯X complex using the experimental value of the parameter. Otherwise, the value of the spectral parameter depends either on the nature of X or on the field strength. In the former case, this parameter is not suitable for measuring the distance. In the latter case, the adduct under field approach is not suitable for analyzing the dependency in question. Then we will calculate the strength of the external field required for the dissociation of the proton from diatomic hydrogen halides, linear N≡C-H and C≡N-H, and nonlinear X_3_C-OH alcohols. If this strength correlates to the p*K*_a_ of the acid, at least within a series of similar molecules, then the effect of the field on the covalent structure of molecules can be neglected in qualitative calculations. Otherwise, the extension of the adduct under field approach to large fields should be justified on a case-by-case basis. Finally, we will analyze the effect of the external field on the geometry of a weakly bound H_3_P=O⋯(HF)_2_ complex, in which the oxygen atom forms two hydrogen bonds simultaneously. Here, we aim to determine the field range at which the structure of this complex changes insignificantly with the exception of the O…H distance.

## 2. Results and Discussion

### 2.1. δ_iso_(^15^N) and ^1^J(^15^N^1^H) as Functions of the N-H Distance in C≡N-H⋯F^−^ and C≡N-H⋯FLi

Recently, we reported the NMR parameters of hydrogen-bonded complexes of the [^13^C≡^15^N]^−^ anion [68]. Of special interest is its complex with hydrogen fluoride. This complex was experimentally studied in a CDF_3_/CDF_2_Cl mixture at 130 K where its structure is C≡N-H⋯F^−^ [69]. This study reported ^1^H, ^15^N, and ^19^F chemical shifts and ^1^J(^1^H^15^N), ^2h^J(^15^N^19^F), and ^h^J(^1^H^19^F) coupling constants. These couplings represent rare examples of spin-spin interactions across hydrogen bonds [70,71,72,73]. It is evident that δ_iso_(^19^F), ^2h^J(^15^N^19^F), and ^h^J(^1^H^19^F) depend on interactions between the fluorine atom and solvent molecules [67]. These interactions are difficult to model, so the calculated and experimental values of these parameters will differ. In contrast, it is reasonable to expect that δ_iso_(^15^N) and ^1^J(^1^H^15^N) depend mainly on the N-H distance, while other interactions affect these parameters only indirectly, through their influence on this distance. In this sense, the ^15^N NMR parameters of C≡N-H can resemble those of pyridines [15,74,75,76].

**Figure 1:**
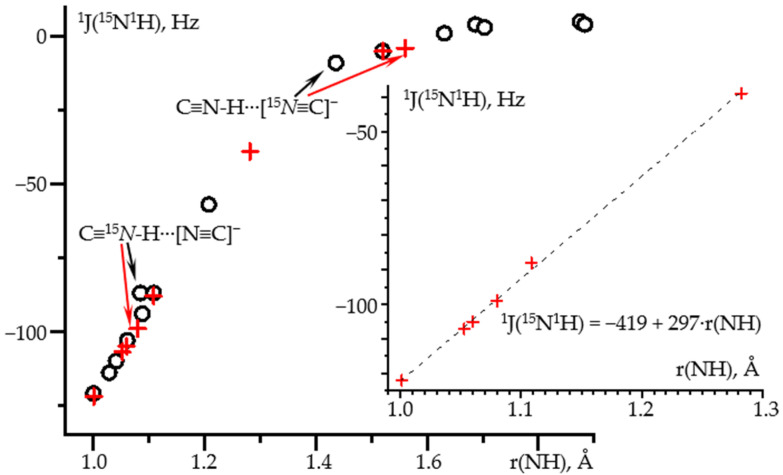
^1^J(^15^N^1^H) as a function of the N-H distance in hydrogen-bonded complexes of C≡^15^N-^1^H obtained at different approximations and PCM = water. Geometry optimization at the wB97XD/def2tzvp and NMR at the GIAO wB97XD/pcJ-2 approximations (black circles). Geometry optimization at the MP2/def2qzvpp and NMR at the GIAO wB97XD/pcJ-3 approximations (red crosses). Insert: the distance range at which this dependence is linear.

Figure 1 shows ^1^J(^15^N^1^H) as a function of the N-H distance in hydrogen-bonded complexes of C≡^15^N-^1^H. The geometry of these complexes was optimized at the wB97XD/def2tzvp approximation and ^1^J(^15^N^1^H) was calculated at the GIAO wB97XD/pcJ-2 approximations. The composition of selected complexes and the corresponding numerical data are collected in Table 1. Other complexes are reported in [68]. The dependence is linear for the distance shorter than 1.3 Å. Small deviations are observed for the proton-bound homodimer of the cyanide anion. The use of more accurate methods, MP2/def2qzvpp and GIAO wB97XD/pcJ-3, corrects these deviations, Figure 1. Note that the geometry of this homodimer depends critically on the approximation used, Table 1. The error in the determination of the geometry, in turn, leads to the deviation in the ^1^J(^15^N^1^H) dependence. Both approaches provide the same functional dependence, where ^1^J(^15^N^1^H) is in Hz and r(NH) stands for the N-H distance in Å and must be shorter than 1.3 Å: ^1^J(^15^N^1^H) = −419 + 297·r(NH).

**Figure 2:**
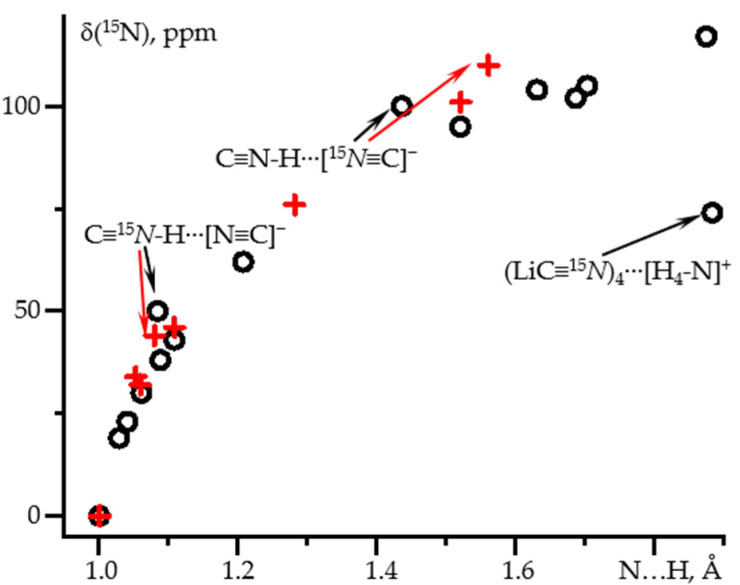
δ_iso_(^15^N) as a function of the N-H distance in hydrogen-bonded complexes of C≡^15^N-^1^H obtained at different approximations and PCM = water. Geometry optimization at the wB97XD/def2tzvp and NMR at the GIAO wB97XD/pcJ-2 approximations (black circles). Geometry optimization at the MP2/def2qzvpp and NMR at the GIAO wB97XD/pcJ-3 approximations (red crosses).

The dependence of δ(^15^N) on the N-H distance in the same complexes is nonlinear, Figure 2 and Table 1. The spread of values is larger than for ^1^J(^15^N^1^H) for both approaches used. However, for distances shorter than 1.3 Å the results are similar. Consequently, in this distance range δ(^15^N) also depends, almost exclusively, on the N-H distance.

**Figure 3:**
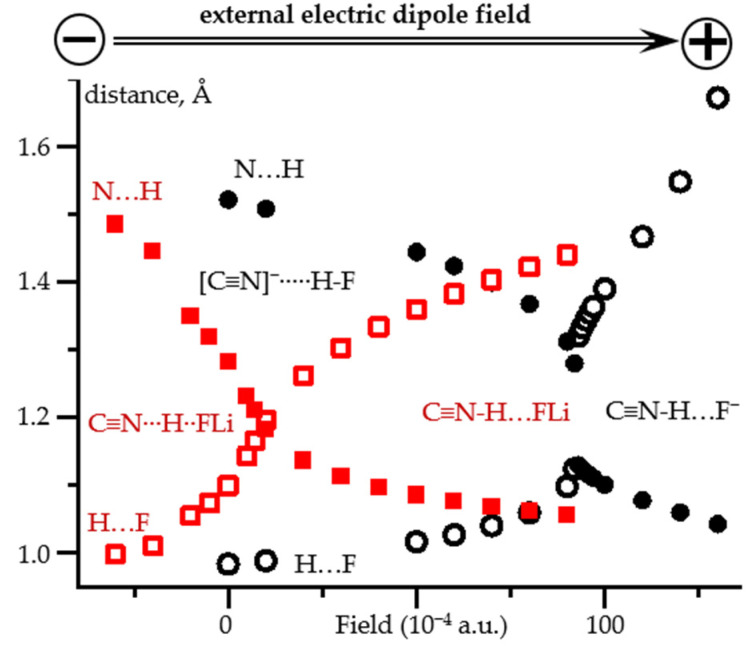
N…H and H…F distances in C≡N-H…F^−^ and C≡N-H…FLi complexes as functions of the applied external electric field at PCM = water. The positive direction of the field corresponds to the direction from the nitrogen to the fluorine atoms. C≡N-H…F^−^: N…H (filled black circles) and H…F (open black circles). C≡N-H…FLi: N…H (filled red squares) and H…F (open red squares).

Now we will check whether it is possible to reproduce these dependencies using only one complex, the geometry of which changes when an external electric field is applied. This will be done for two model complexes C≡^15^N-H⋯F^−^ and C≡^15^N-H⋯FLi. Figure 3 shows the direction of the field and its effect on the geometry of these complexes. Note that the original geometries of these model complexes in the absence of the field are very different. In the first complex the proton is located at the fluorine atom, [C≡N]^−^⋯H-F, Figure 3 and Table S2. In the second complex the proton is shared by the fluorine and nitrogen atoms, [C≡N]^−^⋯H^+^⋯FLi, Figure 3 and Table S2. The qualitative changes in the geometry of these complexes caused by the external electric field are similar. The location of the proton can be changed in both directions by changing the direction of the field. The only principal difference is that in [C≡N]^−^⋯H^+^⋯FLi, this shift occurs smoothly over the entire distance range. In contrast, a transition from [C≡N]^−^⋯H-F to C≡N-H⋯F^−^ occurs abruptly when the field changes from 0.0092 to 0.0093 a.u.

**Figure 4:**
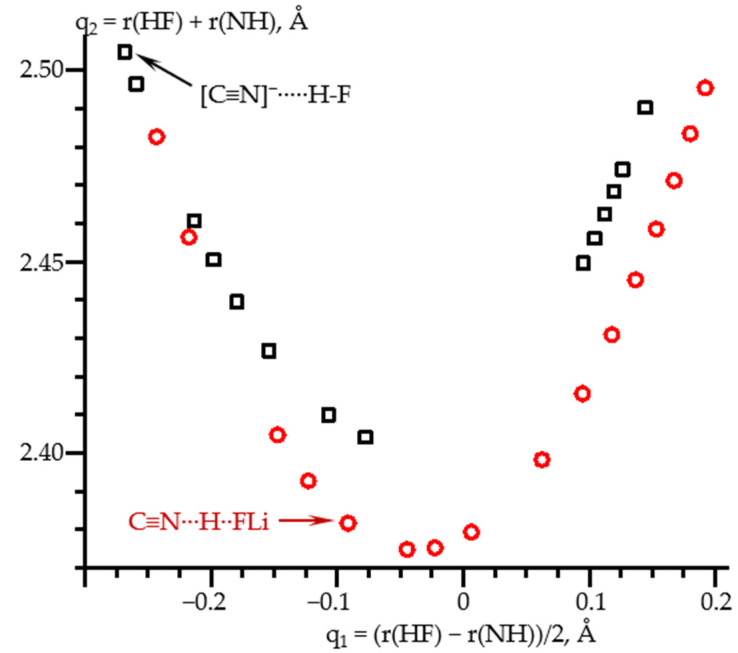
Hydrogen bond correlation q_2_ vs. q_1_ of the calculated equilibrium geometries of C≡N-H…F^−^ (black squares) and C≡N-H…FLi (red circles) complexes at PCM = water and the effect of the external electric field. q_2_ = r(HF) + r(NH) and q_1_ = (r(HF) − r(NH))/2. The equilibrium geometries in the absence of the field are shown by arrows.

These geometric changes are illustrated in Figure 4. q_1_ stands for the distance of the proton with respect to the center of the N…F distance, which is equal to q_2_. The shape of this dependence is the same for all hydrogen bonds [12,77]. Note that for large N…H distances, both dependences coincide, while at large H…F distances they differ. The reason is that in the former area the proton acceptor is the same, [C≡N]^−^, while in the latter they are different, F^−^, and FLi. In all of these limiting cases, the distance between the proton and the donating atom changes only slightly.

**Figure 5:**
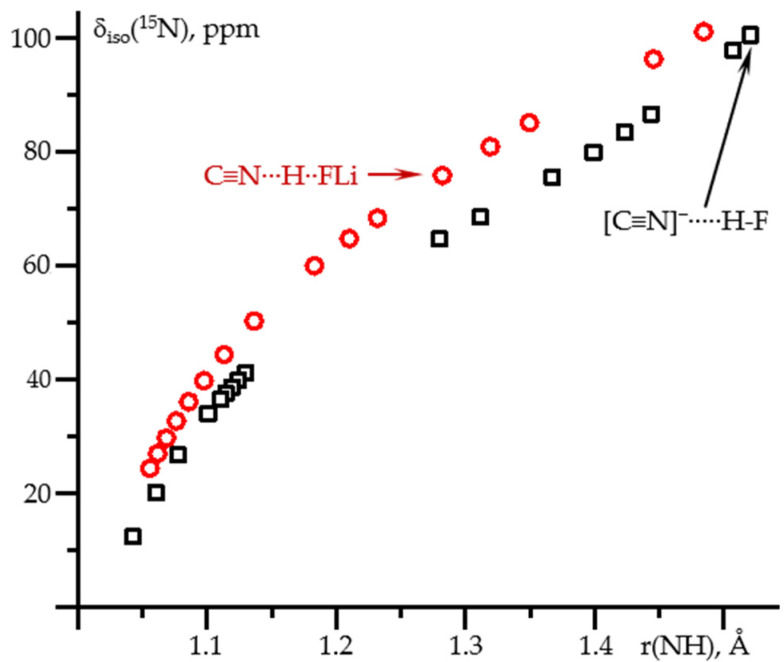
δ_iso_(^15^N) as a function of the N-H distance in C≡N-H…F^−^ (black squares) and C≡N-H…FLi (red circles) complexes at PCM = water and the effect of the external electric field. The values in the absence of the field are shown by arrows.

Figure 5 shows δ_iso_(^15^N) as a function of the N-H distance in these two complexes. At the C≡^15^N-H⋯X and [C≡^15^N]^−^⋯H-X limits (X = F^−^, FLi), δ_iso_(^15^N) is the same for both complexes. These limiting cases correspond to weak hydrogen bonds where the effect of the partner is small. For each of the complexes, the corresponding geometry was achieved at different values of the field. Consequently, the effect of the field on δ_iso_(^15^N) in C≡^15^N-H and [C≡^15^N]^−^ is small and can be neglected. For stronger hydrogen bonds, the area between these limiting cases, δ_iso_(^15^N) depends not only on the N…H distance but also on the partner, Figure 5. Consequently, the spread of δ_iso_(^15^N) values in Figure 2 is not an artifact of the calculations but reflects the difference in the partner. On the other hand, the effect of the partner is small compared to the dependence on the distance, especially at short distances.

**Figure 6:**
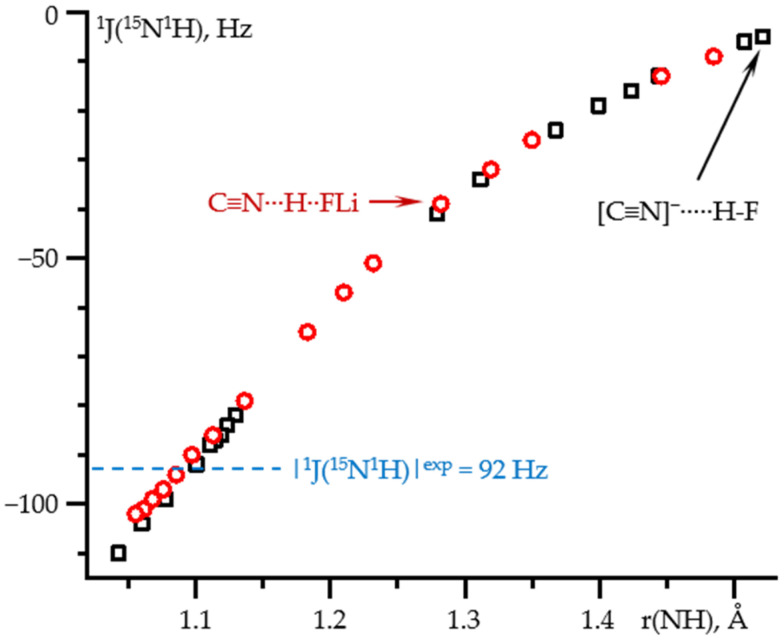
^1^J(^15^N^1^H) as a function of the N-H distance in C≡N-H…F^−^ (black squares) and C≡N-H…FLi (red circles) complexes at PCM = water and the effect of the external electric field. The values in the absence of the field are shown by arrows. The experimental value of ^1^J(^15^N^1^H) in CDF_3_/CDF_2_Cl at 130 K is shown by a blue line [69].

Figure 6 shows ^1^J(^15^N^1^H) as a function of the N-H distance in these two complexes. For both complexes, the dependence is the same over the entire distance range. Consequently, ^1^J(^15^N^1^H) depends exclusively on the N…H distance. Again, for each of the complexes, the given N-H distance was achieved at different values of the field. Consequently, the effect of the field on ^1^J(^15^N^1^H) in any C≡^15^N-^1^H…X complex is small and can be neglected. The experimental value of 92 Hz [69] for a hydrogen-bonded complex of isocyanide with tetrabutylammonium fluoride in CDF_3_/CDF_2_Cl at 130 K corresponds to the N-H distance of 1.095 ± 0.005 Å, Table S2.

### 2.2. Field-Induced Proton Dissociation

**Figure 7:**
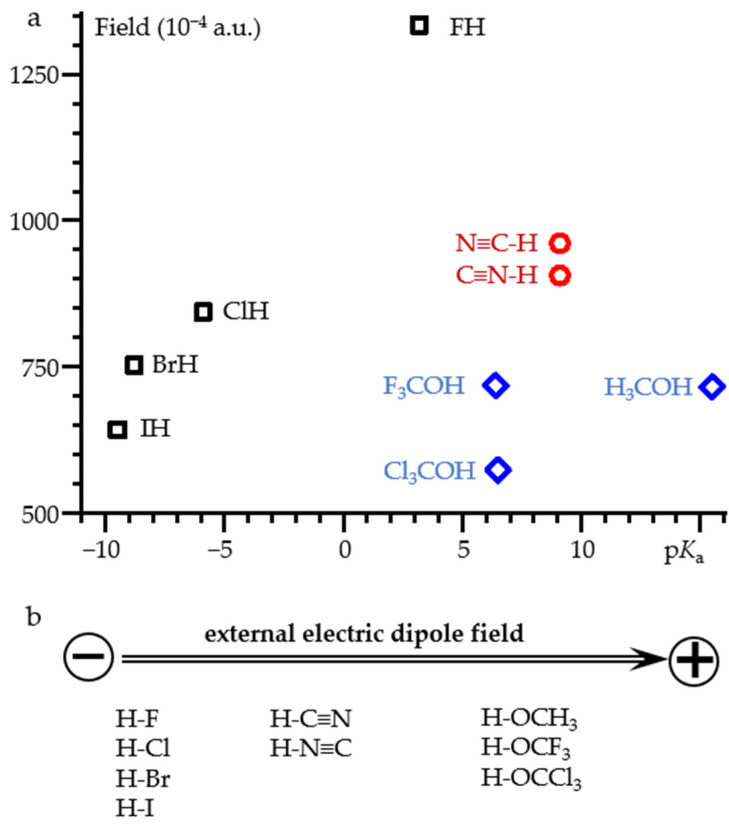
(**a**) The value of the external electric field required for proton dissociation of selected acids as a function of their p*K*_a_. (**b**) The orientation of the acids relative to the direction of the field. For HOCX_3_, the field is directed along the C-O bond.

Figure 7 shows the value of the external electric field required for proton dissociation of selected acids as a function of their p*K*_a_. For diatomic hydrogen halides, the correlation is near linear. The numerical data are reported in Table S3. A noticeable deviation is observed for IH. However, this molecule may require relativistic corrections [78]. Cyanide and isocyanide dissociate at very close field strengths. This, once again, emphasizes the closeness of their properties [68,79,80]. The numerical data are reported in Table S4. For X_3_C-OH (X = H, F, Cl) alcohols, the result depends on whether the field is directed along the C-O bond or along the O-H bond. The numerical data are reported in Tables S5 and S6. In both cases the correlation is absent. Figure 7 shows the case when the field is directed along the C-O bond. When the field is directed along the O-H bond, the limiting value of the field is smaller for all complexes. However, the magnitude of the change depends greatly on the substitute X. First, the X_3_C moieties become asymmetric. This effect is small in H_3_C-OH and the resulting field change is also small. In contrast, F_3_C-OH and Cl_3_C-OH exhibit concerted dissociation of the proton and one of the halogen atoms. Obviously, this behavior has nothing to do with the dissociation of alcohols in water.

Note that the field strength required for proton dissociation is about an order of magnitude higher than that required to simulate the real geometry of hydrogen-bonded complexes in solution. Therefore, the influence of the field on the electronic structure of the molecule as a whole is significant. Let us consider the influence of the field on the geometry of molecules.

### 2.3. Field-Induced Structural Changes

**Figure 8:**
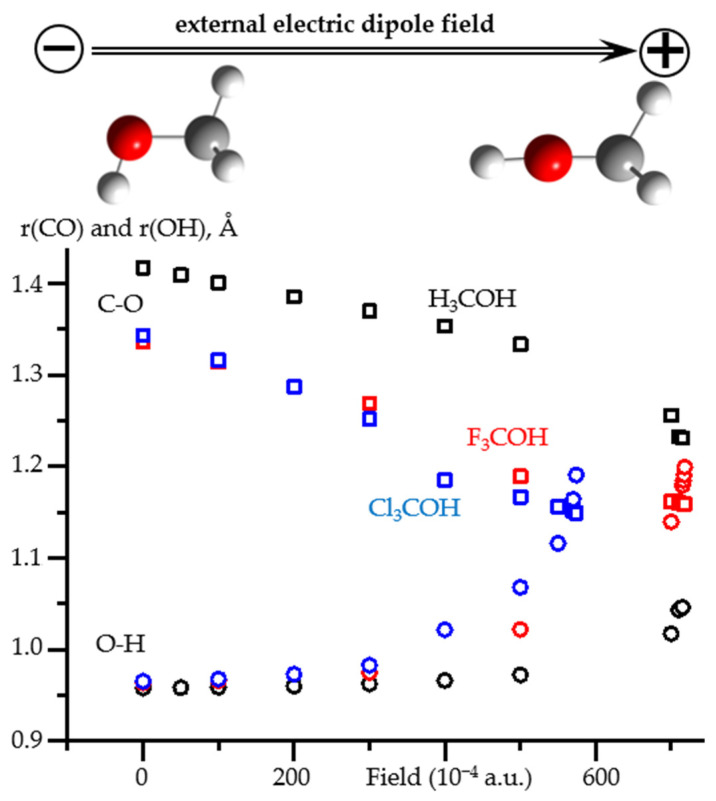
The O-H and C-O distances as functions of an external electric field applied to H_3_COH, F_3_COH, and Cl_3_COH at PCM = water. The O-H distances: H_3_COH (black squares), F_3_COH (red squares), and Cl_3_COH (blue squares). The C-O distances: H_3_COH (black circles), F_3_COH (red circles), and Cl_3_COH (blue circles). The orientation of the acids relative to the direction of the field is shown for H_3_COH.

Figure 8 shows how the C-O and O-H distances in X_3_COH (X = H, F, Cl) alcohols change under the action of the external electric field directed along the C-O bond. The numerical data, including the values of the OCH angle, are reported in Table S5. In the low field range (<0.02 a.u.), only the C-O distance changes noticeably. The changes in the O-H distance and even in the OCH angle are small. In contrast, in the high field range (>0.03 a.u.), the O-H distance increases rapidly and the OCH angle tends to 180°. In the very high field range (>0.05 a.u.), the changes strongly depend on the chemical composition of the molecule. The results obtained at such fields should be treated with caution [66].

On the other hand, the attempt to simulate the effects of solvation using a very strong fictitious electric field is a very rough approximation. Although this approach somehow works for diatomic hydrogen halides, it is difficult to justify it in other cases. In fact, there is no need to use such strong fields. Proton transfer within a hydrogen bond requires much lower fields. The problem is that it is not clear how to apply this approach to systems in which the non-covalent interaction in question is nonlinear or more than one interaction is present.

**Figure 9:**
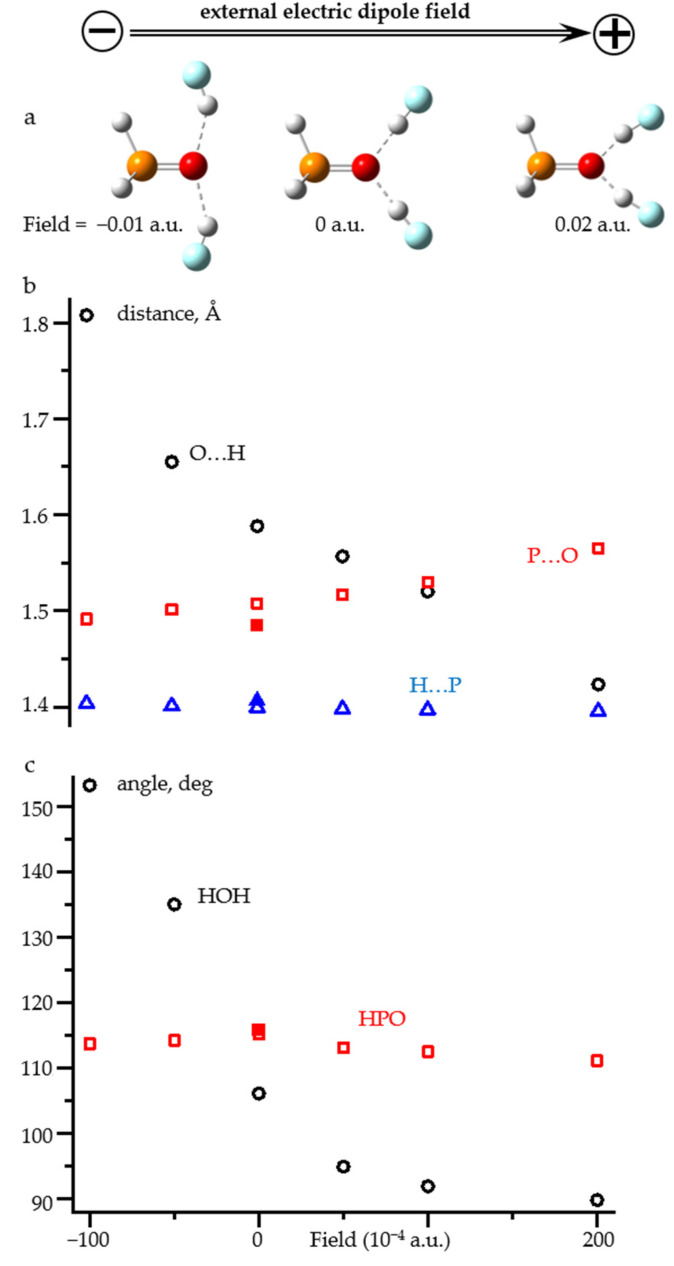
(**a**) Qualitative changes of the structure of H_3_P=O…(H-F)_2_ complex under the action of an external electric field directed along the P=O bond. (**b**) The O…H (open black circles), P=O (open red squares), and H-P (open blue triangles) distances as functions of the field. (**c**) The HOH (open black circles) and HPO (open red squares) angles as functions of the field. The geometric parameters of free H_3_P=O are shown by filled symbols.

Figure 9 shows the changes in the structure of H_3_P=O…(HF)_2_ adduct under the action of an external electric field directed along the P=O bond. The numerical values are reported in Table S7. This hydrogen bond pattern is typical for the P=O group [81,82,83] and anilines [84,85,86]. The O…H distances in this complex vary over a wide range in a narrow field window, Figure 9b. In this field range, the H-P distance remains almost constant. The P=O distance varies, but not very much. The same is true for the H-P=O angle, Figure 9c. Consequently, in the range of small fields (<0.01 a.u.), the effect of the field on the covalent structure of H_3_P=O is small. The effect on the O…H distance is not surprising. However, the applied field significantly changes the hydrogen bond pattern, Figure 9a, and the H…O…H angle, Figure 9b. The effect is strong even in the range of small fields (<0.01 a.u.). Consequently, the adduct under field approach cannot be applied to such complexes without additional geometric constraints. The need for these restrictions qualitatively reduces the value of this approach.

## 3. Materials and Methods

Gaussian 09.D.01 program package (Gaussian, Inc., Wallingford, CT, USA) was used [87]. Geometry optimizations were done in the wB97XD/def2tzvp and MP2/def2qzvpp approximations [88,89,90]. The NMR parameters were calculated using the Gauge-Independent Atomic Orbital (GIAO) method [91] in the wB97XD/pcJ-2, pcJ-3, and aug-pcJ-3 approximations [92,93,94]. All calculations were performed using the polarizable continuum model (PCM) with water as a solvent [95,96,97]. For polar solvents, a change in the values of the dielectric constant has a negligible effect. Therefore, the choice of a specific solvent in the PCM approximation is of no fundamental importance. The default SCRF = PCM method was used to construct the solute cavity.

The wB97XD functional and the pcJ-n basis sets correctly reproduce the experimental values of chemical shielding and scalar spin-spin coupling [43,58,67]. In this work we have converted the calculated ^15^N isotropic chemical shieldings, σ_iso_, to chemical shifts, δ_iso_, δ_iso_ = σ_ref_ − σ_iso_, where σ_ref_ is the isotropic chemical shielding in free C≡N-H in the given approximation. More about this issue can be found elsewhere [98,99,100]. The original σ_iso_(^15^N) are reported in Supplementary Materials.

## 4. Conclusions

This work reports on the response of the geometric and NMR properties of molecular systems to an external electric field. The main issue was the range of field strengths in which this approach can be used to model the geometric and spectral changes experienced by molecular systems in polar media. It has been shown that the main requirement is the presence in the studied molecular system of one and only one bond, the polarizability of which significantly exceeds the polarizability of other bonds. If this requirement is met, then it becomes possible to model even extreme cases. For example, the field required for proton dissociation in hydrogen halides correlates with their p*K*_a_. In contrast, the same correlation is absent for alcohols.

This requirement is met for many complexes with one hydrogen bond. For such complexes, this adduct under field approach can be used to facilitate the analysis of spectral changes associated with geometric changes in the hydrogen bond. This can be conducted in great detail with just two model complexes. For example, in hydrogen-bonded complexes of isocyanide C≡^15^N-^1^H⋯X, in which the N-H distance r(NH) < 1.3 Å, this distance can be estimated from the experimental value of ^1^J(^15^N^1^H):r(NH) = (419 − |^1^J(^15^N^1^H)|)/297 ± 0.01 Å. This correlation does not depend on the chemical nature of X. The chemical shift δ(^15^N) also correlates with r(NH), but it is also slightly influenced by the nature of X.

When the considered molecular system has several bonds with similar polarizabilities and these bonds are not parallel, the response of the system to an external electric field should be evaluated with caution.

In this study, we did not analyze the effect of an external electric field on the energy of complexes. For molecules, this issue was studied in [101]. Note that an intramolecular basis set superposition error can be important [102].

## Figures and Tables

**Table 1:** The ^15^N isotropic chemical shift, δ_iso_(^15^N), referenced to free C≡^15^N-^1^H at 0 ppm and the ^15^N-^1^N scalar coupling constant, ^1^J(^15^N^1^H), as functions of the N-H distance in hydrogen-bonded complexes of C≡^15^N-^1^H obtained at different approximations and PCM = water.

Adduct	^1^Structure: wB97XD/def2tzvp NMR: wB97XD/pcJ-2			Structure: MP2/def2qzvpp NMR: wB97XD/pcJ-3		
	r(NH), Å	δ_iso_(^15^N), ppm	^1^J(^15^N^1^H), Hz	r(NH), Å	δ_iso_(^15^N), ppm	^1^J(^15^N^1^H), Hz
C≡^15^N-^1^H	1.0007	0 (^2^77.6)	−121	1.0010	0 (^2^74.3)	−122
C≡^15^N-^1^H⋯Cl^−^	-	-	-	1.0528	34	−107
(C≡^15^N-^1^H)_3_⋯F^−^	1.0612	30	−103	1.0599	32	−105
C≡^15^*N*-^1^H⋯[^15^N≡C]^−^	1.0847	50	−87	1.0800	44	−99
(C≡^15^N-^1^H)_2_⋯F^−^	1.1086	43	−87	1.1086	46	−88
C≡^15^N-^1^H⋯FLi	1.2079	62	−57	1.2821	76	−39
C≡^15^N-^1^H⋯F^−^	1.5202	95	−5	1.5208	101	−5
C≡^15^N-^1^H⋯[^15^*N*≡C]^−^	1.4362	100	−9	1.5609	110	−4
[^15^N≡C]^−^	-	124	-	-	129	-

^1^ Data from [68]. ^2^ The ^15^N isotropic chemical shielding, σ_iso_(^15^N).

## Data Availability

The data presented in this study are available in Supplementary Materials.

## Supplementary Materials

The following are available online, Table S1: The N-H distance in hydrogen-bonded complexes of C≡^15^N-^1^H, the ^15^N isotropic chemical shielding, σ_iso_(^15^N), and the ^15^N-^1^N scalar coupling constant, ^1^J(^15^N^1^H), obtained at different approximations and PCM = water. Table S2: The H…F and N-H distances, the ^15^N isotropic chemical shielding, σ_iso_(^15^N), and the ^15^N-^1^N scalar coupling constant, ^1^J(^15^N^1^H), in C≡^15^N-^1^H⋯F^−^, and C≡^15^N-^1^H⋯FLi as functions of the external electric field under the PCM = water approximation. Geometry optimization: MP2/def2qzvpp. NMR calculations: GIAO, wB97XD/pcJ-3. Table S3: The F-H, Cl-H, Br-H, and I-H distances in HF, ClH, BrH, and IH as functions of the external electric field under the PCM = water approximation. Geometry optimization: wB97XD/def2tzvp. Table S4: The C-H and N-H distances in NCH and CNH as functions of the external electric field under the PCM = water approximation. Geometry optimization: wB97XD/def2tzvp. Table S5: The O-H and C-O distances and the COH angle in H_3_COH, F_3_COH, and Cl_3_COH, as functions of the external electric field directed along the C-O bond under the PCM = water approximation. Geometry optimization: wB97XD/def2tzvp. Table S6: The O-H and C-O distances and the COH angle in H_3_COH, F_3_COH, and Cl_3_COH, as functions of the external electric field directed along the O-H bond under the PCM = water approximation. Geometry optimization: wB97XD/def2tzvp. Table S7: The O…H, P=O, and H-P distances and HOH, POH, and HPO angles in H_3_P=O…(HF)_2_ as functions of the external electric field under the PCM = water approximation. Geometry optimization: wB97XD/def2tzvp.
