# Supplementary material for: Modeling of the Response of Hydrogen Bond Properties on an External Electric Field: Geometry, NMR Chemical Shift, Spin-Spin Scalar Coupling
Source: Molecules. 2021 Aug 18;26(16):4967. doi: 10.3390/molecules26164967 (PMC8399935; doi:10.3390/molecules26164967)
Supplement: Supplementary file 1 [file molecules-26-04967-s001.zip › molecules-1318281-supplementary.pdf]

Supporting Information for Publication

Modeling of the response of hydrogen bond properties on an external electric field: geometry, NMR chemical shift, spin-spin scalar coupling.

Ilya G. Shenderovich [1,2,*], Gleb S. Denisov [2]

[1] Institute of Organic Chemistry, University of Regensburg, Universitaetstrasse 31, 93053 Regensburg, Germany; Ilya.Shenderovich@ur.de
[2] Department of Physics, St. Petersburg State University, 198504 St. Petersburg, Russia; gldenisov@yandex.ru
* Correspondence: Ilya.Shenderovich@ur.de

Table S1. The N-H distance in hydrogen bonded complexes of C≡^{15}N-^{1}H, the ^{15}N isotropic chemical shielding, σ_{iso}(^{15}N), and the ^{15}N-^{1}N scalar coupling constant, ^{1}J(^{15}N^{1}H), obtained at different approximations and PCM = water.

Adduct	Structure: MP2/def2qzvpp	NMR: wB97XD/pcJ-3		NMR: wB97XD/aug-pcJ-3	
	r(NH), Å	σ_{iso}(^{15}N), ppm	^{1}J(^{15}N^{1}H), Hz	σ_{iso}(^{15}N), ppm	^{1}J(^{15}N^{1}H), Hz
C≡^{15}N-^{1}H	1.0010	74.3	−122	74.3	−122
C≡^{15}N-^{1}H···Cl^{-}	1.0528	40.0	−107	-	-
(C≡^{15}N-^{1}H)$_3$···F^{-}	1.0599	42.4	−105	-	-
C≡^{15}*N*-^{1}H···[^{15}N≡C]$^{-}$	1.0800	30.4	−99	-	-
(C≡^{15}N-^{1}H)$_2$···F^{-}	1.1086	28.4	−88	-	-
C≡^{15}N-^{1}H···FLi	1.2821	−1.5	−39	−1.5	−39
C≡^{15}N-^{1}H···F^{-}	1.5208	−26.2	−5	-	-
C≡^{15}N-^{1}H···[^{15}*N*≡C]$^{-}$	1.5609	−36.1	−4	-	-
[^{15}N≡C]$^{-}$	-	−54.9	-	-	-

Table S2. The H…F and N-H distances, the ^{15}N isotropic chemical shielding, $\sigma_{iso}(^{15}N)$, and the ^{15}N-^{1}N scalar coupling constant, $^{1}J(^{15}N^{1}H)$, in C≡^{15}N-^{1}H···F^{-}, and C≡^{15}N-^{1}H···FLi as functions of the external electric field under the PCM = water approximation. Geometry optimization: MP2/def2qzvpp. NMR calculations: GIAO, wB97XD/pcJ-3.

[1]Field, 10^{-4} a.u.	C≡^{15}N-^{1}H···F^{-} r(H···F), Å	r(NH), Å	$\sigma_{iso}(^{15}N)$, ppm	$^{1}J(^{15}N^{1}H)$, Hz	C≡^{15}N-^{1}H···FLi r(H···F), Å	r(NH), Å	$\sigma_{iso}(^{15}N)$, ppm	$^{1}J(^{15}N^{1}H)$, Hz
−30	-	-	-	-	0.9981	1.4845	−26.8	−9
−20	-	-	-	-	1.0107	1.4457	−22.0	−13
−10	-	-	-	-	1.0551	1.3496	−10.8	−26
−5	-	-	-	-	1.0735	1.3192	−6.6	−32
0	0.9840	1.5208	−26.2	−5	1.0995	1.2821	−1.5	−39
5	-	-	-	-	1.1428	1.2319	6.0	−51
7	-	-	-	-	1.1652	1.2100	9.6	−57
10	0.9890	1.5074	−23.6	−6	1.1962	1.1831	14.4	−65
20	-	-	-	-	1.2617	1.1365	24.1	−79
30	-	-	-	-	1.3024	1.1131	30.0	−86
40	-	-	-	-	1.3334	1.0975	34.6	−90
44	-	-	-	-	1.3443	1.0924	36.1	−92
45	-	-	-	-	1.3469	1.0912	36.5	−92
46	-	-	-	-	1.3495	1.0900	36.9	−93
50	1.0168	1.4438	−12.3	−13	1.3595	1.0857	38.3	−94
60	1.0272	1.4233	−9.1	−16	1.3823	1.0762	41.7	−97
70	1.0405	1.3990	−5.5	−19	1.4029	1.0683	44.7	−99
80	1.0594	1.3673	−1.2	−24	1.4219	1.0616	47.4	−101
90	1.0984	1.3115	5.8	−34	1.4397	1.0557	50.0	−102
92	1.1245	1.2796	9.7	−41	-	-	-	-
93	1.3199	1.1297	33.2	−82	-	-	-	-
94	1.3323	1.1238	34.5	−84	-	-	-	-
95	1.3435	1.1189	35.7	−86	-	-	-	-
96	1.3538	1.1145	36.8	−87	-	-	-	-
97	1.3633	1.1107	37.8	−88	-	-	-	-
99	1.3810	1.1039	39.6	−91	-	-	-	-
100	1.3895	1.1008	40.5	−92	-	-	-	-
101	1.3977	1.0980	41.3	−93	-	-	-	-
110	1.4663	1.0777	47.7	−99	-	-	-	-
120	1.5473	1.0604	54.3	−104	-	-	-	-
130	1.6712	1.0429	62.1	−110	-	-	-	-

[1]The positive direction of the external field is from the negative to positive charge.

Table S3. The F-H, Cl-H, Br-H, and I-H distances in HF, ClH, BrH, and IH as functions of the external electric field under the PCM = water approximation. Geometry optimization: wB97XD /def2tzvp.

[1]Field, 10^{-4} a.u.	HF, Å	HCl, Å	HBr, Å	HI, Å
0	0.9217	1.2815	1.4213	1.6100
25	0.9226	1.2828	-	-
50	0.9236	1.2842	-	-
75	0.9246	1.2858	-	-
100	0.9256	1.2875	-	-
125	0.9267	1.2893	-	-
150	0.9279	1.2913	-	-
200	0.9303	-	-	-
300	0.9357	-	-	-
400	0.9419	-	-	-
500	-	1.3389	-	-
600	-	-	-	1.7648
630	-	-	-	1.8123
640	-	-	-	1.8499
643	-	-	-	1.8835
700	-	-	1.5853	
750	-	-	1.6674	
753	-	-	1.6883	
800	-	1.4564		
830	-	1.4883		
840	-	1.5037		
844	-	1.5111		
1000	1.0082			
1300	1.1140			
1300	1.1560			
1333	1.1669			
1334	1.1735			

[1]The positive direction of the external field is from the negative to positive charge.

Table S4. The C-H and N-H distances in NCH and CNH as functions of the external electric field under the PCM = water approximation. Geometry optimization: wB97XD /def2tzvp.

[1]Field, 10^{-4} a.u.	NCH r(CH), Å	CNH r(NH), Å
0	1.0706	1.0007
50	1.0732	1.0038
100	1.0762	1.0073
150	1.0795	1.0111
200	1.0832	1.0154
300	1.0919	1.0251
400	1.1027	1.0370
500	1.1162	1.0518
600	1.1332	1.0706
700	1.1553	1.0960
800	1.1864	1.1343
900	1.2393	1.2281
905	-	1.2485
906	-	1.2576
950	1.3019	
960	1.3385	
961	1.3483	

[1]The positive direction of the external field is from the negative to positive charge.

Table S5. The O-H and C-O distances and the COH angle in H_3COH, F_3COH, and Cl_3COH, as functions of the external electric field directed along the C-O bond under the PCM = water approximation. Geometry optimization: wB97XD /def2tzvp.

[1]Field, 10^{-4} a.u.	H_3COH			F_3COH			Cl_3COH		
	r(OH), Å	COH, deg.	r(CO), Å	r(OH), Å	COH, deg.	r(CO), Å	r(OH), Å	COH, deg.	r(CO), Å
0	0.9580	107.8	1.4173	0.9637	109.9	1.3359	0.9652	110.1	1.3432
50	0.9583	108.9	1.4090	-	-	-			
100	0.9588	110.1	1.4010	0.9657	113.1	1.3141	0.9680	113.8	1.3162
200	0.9602	112.5	1.3855	-	-	-	0.9730	118.8	1.2872
300	0.9625	115.2	1.3700	0.9750	122.6	1.2688	0.9829	127.9	1.2519
400	0.9662	118.4	1.3532	-	-	-	1.0217	180.0	1.1851
500	0.9724	122.8	1.3333	1.0220	175.2	1.1892	1.0679	180.0	1.1666
550	-	-	-	-	-	-	1.1161	180.0	1.1564
570	-	-	-	-	-	-	1.1643	180.0	1.1509
574	-	-	-	-	-	-	1.1908	180.0	1.1491
700	1.0173	149.5	1.2560	1.1397	180.0	1.1619			
710	1.0434	174.3	1.2327	1.1618	180.0	1.1604			
715	1.0463	176.4	1.2312	1.1796	180.0	1.1595			
716				1.1847	180.0	1.1593			
717				1.1908	180.0	1.1591			
718				1.1992	180.0	1.1588			

[1]The positive direction of the external field is from the negative to positive charge.

Table S6. The O-H and C-O distances and the COH angle in H_3COH, F_3COH, and Cl_3COH, as functions of the external electric field directed along the O-H bond under the PCM = water approximation. Geometry optimization: wB97XD /def2tzvp.

[1]Field, 10^{-4} a.u.	H_3COH r(OH), Å	H_3COH COH, deg.	H_3COH r(CO), Å	F_3COH r(OH), Å	F_3COH COH, deg.	F_3COH r(CO), Å	Cl_3COH r(OH), Å	Cl_3COH COH, deg.	Cl_3COH r(CO), Å
0	0.9580	107.8	1.4173	0.9637	109.9	1.3359	0.9652	110.1	1.3432
100	-	-	-	-	-	-	0.9703	111.9	1.3326
200	-	-	-	-	-	-	0.9763	113.9	1.3059
244	-	-	-	-	-	-	0.9811	114.3	1.2937
300	-	-	-	0.9813	117.8	1.297			
400	-	-	-	0.9939	121.0	1.2719			
410	-	-	-	0.9958	121.2	1.2679			
413	-	-	-	0.9967	121.2	1.2664			
500	0.9837	101.8	1.4061						
600	0.9948	102.1	1.3916						
620	0.9987	102.8	1.3832						
625	0.9998	103.0	1.3802						
630	1.0013	103.4	1.3762						
634	1.0032	103.9	1.3702						

[1]The positive direction of the external field is from the negative to positive charge.

Table S7. The O…H, P=O, and H-P distances and HOH, POH, and HPO angles in $H_3P{=}O\ldots(HF)_2$ as functions of the external electric field under the PCM = water approximation. Geometry optimization: wB97XD /def2tzvp.

[1]Field, 10^{-4} a.u.	r(O…H), Å	r(PO), Å	r(HP), Å	HOH, deg.	POH, deg.	HPO, deg.
−100	1.8079	1.4913	1.4032	153.2	103.4	113.7
−50	1.6552	1.5011	1.4007	135.0	112.5	114.2
0	1.5882	1.5073	1.3989	106.1	127.0	115.1
50	1.5567	1.5168	1.3976	94.9	132.5	113.1
100	1.5200	1.5297	1.3965	91.9	134.1	112.5
200	1.4233	1.5652	1.3951	89.8	135.1	111.1
0	$H_3P{=}O$	1.4849	1.4066	-	-	115.8

[1]The positive direction of the external field is from the negative to positive charge.
